# Relationship of Cardiorespiratory Fitness and Body Mass Index with the Incidence of Dyslipidemia among Japanese Women: A Cohort Study

**DOI:** 10.3390/ijerph16234647

**Published:** 2019-11-22

**Authors:** Takahisa Ohta, Junzo Nagashima, Hiroyuki Sasai, Naokata Ishii

**Affiliations:** 1 Department of Life Sciences, Graduate School of Arts and Sciences, The University of Tokyo, 3-8-1 Komaba, Meguro-ku, Tokyo 153-8902, Japan; hiroyuki.sasai@gmail.com (H.S.); ishii@idaten.c.u-tokyo.ac.jp (N.I.); 2 Yokohama Sports Medical Center, Nissan Stadium, 3302-5, Kodukue-chou, Kouhoku-ku, Yokohama-City 222-0036, Japan; ju01-nagashima@yspc.or.jp; 3 Division of Cardiology, Department of Internal Medicine, St. Marianna University School of Medicine, 2-16-1, Sugao, Miyamae-ku, Kawasaki 216-8511, Japan

**Keywords:** dyslipidemia, cardiorespiratory fitness, body mass index, low-density lipoprotein, high-density lipoprotein, cholesterol, aerobic exercise

## Abstract

Low cardiorespiratory fitness (CRF) and obesity are independent risk factors for dyslipidemia. We investigated the synergistic effects of CRF and obesity on the incidence of dyslipidemia among Japanese women. Of 7627 participants, 927 normolipidemic Japanese women completed a submaximal exercise test, medical examination, and a questionnaire on smoking and alcohol drinking. The incidence of dyslipidemia was defined as having at least one of the following: high-density lipoprotein cholesterol < 40 mg/dL, low-density lipoprotein cholesterol ≥ 140 mg/dL, fasting triglyceride ≥ 150 mg/dL, or physician-diagnosed dyslipidemia. Multivariable hazard ratios (HRs) and 95% confidence intervals (CI) were calculated using a Cox proportional hazard regression model. During the follow-up period of ≤16 years (median 1 year), 196 (21.1%) women developed dyslipidemia. Compared with those in the body mass index (BMI)-specific (< or ≥25.0 kg/m^2^) lowest CRF tertile, the multivariable HRs for dyslipidemia in the highest CRF tertile were 1.36 (95% CI, 0.75–2.48) for women with BMI ≥ 25 kg/m^2^ and 0.70 (95% CI, 0.45–1.09) for those with BMI < 25 kg/m^2^ (*p* < 0.01 for interaction). These results suggest that CRF and BMI are interdependent and, together, they affect the incidence of dyslipidemia among Japanese women. CRF is inversely related to a lower incidence of dyslipidemia with low BMI.

## 1. Introduction

According to the Vital Statistics of Japan 2017 [1], cardiovascular disease (CVD) is the second leading cause of deaths in Japan, and its incidence rate has steadily increased over the past 40 years. As elevated levels of total cholesterol (TC) [2], low-density lipoprotein cholesterol (LDL-C) [3], and triglycerides (TG) [4] and low high-density lipoprotein cholesterol (HDL-C) [5] have been established as cardiovascular risk factors, countermeasures for dyslipidemia are necessary to prevent CVD. In addition to genetic and environmental factors, lifestyle-related factors, such as weight control and physical inactivity, are associated with the development of dyslipidemia, whereas proper diet and physical activity are believed to delay or prevent its onset [6,7].

Although both aerobic exercise (AE) and resistance exercises effectively improved lipid metabolism, the former is recommended for wider generations because it is simple and sufficiently increases energy expenditure [8,9]. However, systematic reviews based on randomized controlled trials (RCTs) found that the effectiveness of AE on TC and HDL-C levels were inconclusive [10,11,12,13,14].

This inconsistency could be affected by wide variations in body size (as measured by body mass index [BMI]), which is another established risk factor for dyslipidemia [15,16]. From a mechanistic viewpoint, obesity-induced insulin resistance plays an important role in blunting adiponectin secretion [17] and promoting tumor necrosis factor-α secretion in the adipose tissues, which eventually leads to increased lipid production in the liver [18,19].

However, whether the beneficial associations of CRF, a partial reflection of AE [20], with dyslipidemia could be modified by the obesity level remains unclear. In addition, the associations of CRF and BMI with blood lipid profiles have not been well-studied on Japanese adults [21], and only a few studies [22] on Japanese women currently exist. Therefore, accumulating epidemiological evidence in this research field is warranted. Hence, we aim to investigate associations of combined CRF and BMI with the risk of developing dyslipidemia among Japanese women.

## 2. Methods

### 2.1. Participants

The participants visited the Yokohama Sports Medical Center to receive a sports program service (SPS), which included a medical check-up and physical fitness test. The SPS was first introduced in April 1998, in order to improve the health of civil residents inside and/or outside the Yokohama city. Participants could find the information about the SPS at the center’s website or public information issued by the Yokohama city to which they voluntarily applied for the service. Approximately 1500 people visited the center to receive the SPS annually at an average of 10 visitors per day. The consultation fee is 15,000 JPY (approximately $137 in 2019) for participants living or working inside the city and 7500 JPY ($69) for those aged ≥65 years old, whereas for other visitors, the fee is 17,000 JPY ($155) and 8500 JPY ($77) for those aged ≥65 years old. This study included 7627 women who received the SPS between April 1998 and July 2016. We excluded participants with a history of dyslipidemia, medication of dyslipidemia, and abnormal lipid profile of either HDL < 40 mg/dL, LDL ≥ 140 mg/dL, or TG ≥ 150 mg/dL. We then excluded participants with abnormal electrocardiography findings at rest and during a physical exercise stress test, and who had limited physical exercise stress data because of failure to complete the test due to extreme fatigue. 

All participants provided written informed consent for the SPS and potential data usage for research purposes, including this study. The study was conducted in accordance with the Declaration of Helsinki, and the protocol was approved by the Research Ethics Committee of Yokohama City Sports Medical Center (Project identification code 2017-01).

### 2.2. Medical Examination

Height and body weight were measured using the height–weight scale (WB-510, Tanita Co., Tokyo, Japan) when participants stood barefoot. BMI was calculated as body weight in kilograms divided by squared height in meters (kg/m^2^). The resting blood pressure was measured using the Riva–Rocci–Korotkov method (mercury sphygmomanometer) after 5 min of rest with participants in the sitting position. Pulse pressure was computed as systolic blood pressure (SBP) minus diastolic blood pressure. Blood was collected after a 12-h fasting period, and blood levels of TC, HDL-C, LDL-C, and TG were measured using Roche INTEGRA 400 plus (Roche International Ltd., Basel, Switzerland). A self-reported questionnaire was administered to collect information on the following parameters: alcohol drinking (yes/no), smoking (yes/no), medication of dyslipidemia (yes/no).

### 2.3. Physical Exercise Stress Test

We adopted a physical working capacity at 75% of the maximum heart rate (PWC75% HR Max) as the CRF indicator [23], using submaximal graded exercise test methods on an electronic bicycle ergometer (The Multi Exercise Test System, ML-1800, Fukuda-Denshi, Tokyo, Japan). During the graded exercise test, the rate of increase in load (10–60 watts/min), which is an individualized ramp protocol, was determined based on AE habits of the participants by a comprehensive judgment of experts. The target heart rate was set at ≥75% of the estimated maximum heart rate (220−age) and the test was stopped when the target heart rate was achieved. In addition, we stopped the test when an abnormal electrocardiogram (ST depression and frequent occurrence of extrasystole) was identified by cardiologists or participants were unable to pedal with regular rhythm (50 rpm) and complained of physical deconditioning. The participants completed the exercise test in approximately 10 min.

### 2.4. Assessment of Dyslipidemia

The incidence of dyslipidemia was defined based on the Japan Atherosclerosis Society published in 2012 [24] (high-density lipoprotein cholesterol < 40 mg/dL, low-density lipoprotein cholesterol ≥ 140 mg/dL, fasting triglyceride ≥ 150 mg/dL) and physician-diagnosed dyslipidemia during the follow-up.

### 2.5. Statistical Analysis

We first divided all participants into approximate 10-year age strata (18–29, 30–39, 40–49, 50–59, 60–69, and ≥70). Within each of the age strata, we categorized the participants into identical-sized tertiles based on their CRF level. Finally, we recombined the six age-stratified CRF tertiles into one and created a dataset with age-adjusted CRF tertiles (lowest, middle, and highest) of all participants. Person-years were calculated as the total sum of each individual’s follow-up period. We performed a one-way analysis of variance or chi-square test to compare baseline characteristics. Continuous variables are presented as mean and standard deviation (SD), and categorical variables as percentages.

We applied a Cox proportional hazard model to assess associations between the CRF and incidence of dyslipidemia with adjustments of potential confounding factors, such as SBP, smoking, alcohol drinking, and BMI [24]. Multivariable hazard ratios (HRs) and 95% confidence intervals (CI) were calculated using the lowest CRF tertile as a reference category. We also entered continuous CRF variables into a separate Cox model and tested its linearity. To assess the association of BMI with incident dyslipidemia, we repeated the same analyses using the BMI category (< or ≥25.0 kg/m^2^) as an exposure. Similarly, age, SBP, alcohol drinking, smoking, and CRF were statistically adjusted in these models.

To analyze the association of CRF and BMI with the incidence of dyslipidemia, we first stratified the participants by BMI (i.e., < or ≥25.0 kg/m^2^), and then tested the association of CRF with the development of dyslipidemia in two separate models. We also confirmed the existence of effect modification using the interaction term (CRF tertiles and BMI category) in the combined model. SPSS Statistics version 25 (IBM-SPSS, Inc., Chicago, IL, USA) was used to perform all the statistical analyses. A *p*-value < 0.05 was considered statistically significant.

## 3. Results

**Figure 1:**
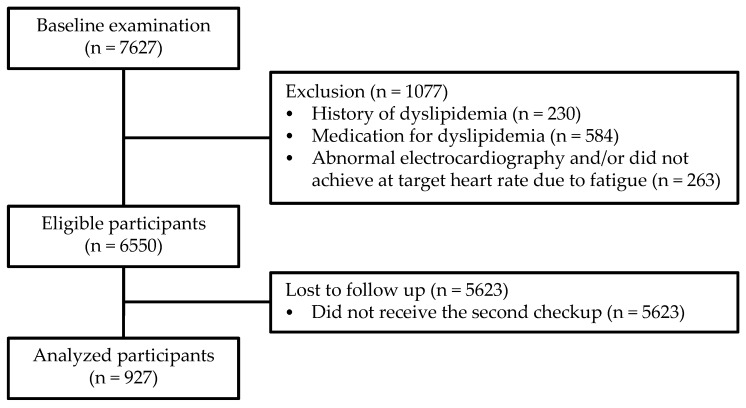
Flowchart of participant selection.

Between April 1998 and July 2016, 7627 women underwent the baseline measurements. Of these, 1077 participants were screened out due to the exclusion criteria, and 5623 participants were lost to the follow-up. Finally, only 927 women aged 18 to 92 years formed the final analysis set (Figure 1). At baseline, the mean age (SD) was 48.9 (16.2) years. During the follow-up period of 16 years (median 1.0 year, maximum 16.0 years), 196 (21.1%) developed dyslipidemia. The number of incidences per 1000 person-years was 117.9.

Table 1 shows the characteristics of participants at baseline according to the CRF tertiles. When the CRF was higher, the BMI and SBP tended to be lower. However, no significant difference was observed based on the prevalence of smokers but not drinkers between the groups.

Table 2 shows multivariable HRs and 95% CI of the incidence of dyslipidemia according to the CRF tertiles and BMI category (< or ≥25.0 kg/m^2^). There was an inverse dose–response relationship between CRF and the risk of dyslipidemia in the age-adjusted model (*p* for trend = 0.006). A significant inverse relationship was retained even after adjustment for SBP, alcohol drinking, and smoking (*p* for trend *=* 0.017). However, adding BMI into the model as a covariate diminished this significance. In contrast, the risk of dyslipidemia was 2.54 times greater in women with a BMI ≥ 25.0 kg/m^2^ than those with a BMI < 25.0 kg/m^2^ in the fully adjusted model.

In Table 3, we presented the BMI-stratified, combined analyses on the associations of CRF and BMI with the risk of developing dyslipidemia. In the normal weight category, linear trends were observed in both age-adjusted and multivariable models (*p* for trend = 0.09 and 0.11, respectively). The associations of CRF with the risk of dyslipidemia were significantly greater for women with a BMI < 25 kg/m^2^ compared with those with a BMI of ≥25.0 kg/m^2^ (*p* < 0.01 for interaction). In the combined analysis based on the lowest CRF tertile among women with BMI ≥ 25 kg/m^2^ as a reference, the risks of incident dyslipidemia were 50% or lower in the middle and highest CRF tertiles of women with BMI < 25 kg/m^2^ than the reference category.

## 4. Discussion

In this study, we analyzed 927 Japanese women during a follow-up period of up to 16 years (median, 1 year) and investigated the associations of BMI and CRF, as a partial reflection of physical activity [25,26], with the incidence of dyslipidemia. The results suggest that non-obese (BMI < 25 kg/m^2^) women with a higher CRF had a lower incidence of dyslipidemia, and the benefits of high CRF were less emphasized in obese women. These also suggest that sustaining high CRF partly by regular physical activity may lower the risk of developing dyslipidemia in non-obese Japanese women. However, its influence may be suppressed in obese Japanese women.

Kelly et al. reviewed 22 RCTs that examined the influence of walking (a representative form of moderate-intensity AE) intervention on TC and HDL-C and revealed that AE decreased non-HDL-C by 4% (5.6 mg/dL) [27]. This meta-analysis indicated that moderate AE can possibly prevent the development of dyslipidemia. An observational cohort study among Japanese men reported an inverse dose–response association between CRF and elevated non-HDL-C. This study also showed that men with high CRF exhibited an approximately 20% lower risk of dyslipidemia as compared to those with low CRF [21]. Furthermore, other observational studies on women reported lower CRF as a risk factor for all-cause mortality and CVD-associated mortality [28,29,30,31] and metabolic syndrome [32]. Lyerly et al. reported a longitudinal study that examined the relationship among CRF, BMI, and all-cause mortality, including CVD, and demonstrated that overweight women had a 1.85 times (fit group) and 2.26 times (unfit group) higher mortality rate than the normal weight and higher CRF population, respectively [33]. These prior studies generally support our study findings, although our participants were Japanese women, and the outcome of interest was the incidence of dyslipidemia. 

The observation that non-obese women had a greater benefit of high CRF for preventing dyslipidemia could be supported by recent re-analytic findings from the STRRIDE trial [34]. The trial is a multicenter RCT testing the effects of AE amount and intensity on CVD risk factors among overweight or obese women with mild dyslipidemia. Swift et al. demonstrated that AEs elicited no significant improvements in lipid profiles and insulin sensitivity among women without weight loss (<3% from their initial weight) irrespective of AE intensity and amount [35]. Although CRF is a well-accepted independent predictor for the development of CVDs, the benefit of high CRF might be largely masked in obese women who already have an increased risk of dyslipidemia.

Regarding the strengths of our study, this was the first study investigating the relationship of BMI (an index of obesity) and CRF (a partial reflection of physical activity) with the incidence of dyslipidemia among Japanese women after a similar study was conducted among children and adolescents [36]. In contrast, there are several limitations in this study. First, we measured the CRF with a submaximal exercise test that has lower precision than a maximal exercise test. However, the estimated maximum oxygen uptake used in this study was highly comparable with maximal measurements [37,38]. Thus, the risk of misclassification when dividing the participants into the CRF tertiles would not be large. Second, the CRF was evaluated using a one-time “snapshot” measurement at baseline, not repeated measurements over a long period of time. Longitudinal changes in CRF during the follow-up period could be considered a dilution bias, and this might weaken the relationship between CRF and the incidence of dyslipidemia. Third, since approximately half of the variations in CRF are attributed to the genotypes [39], CRF may not be a good indicator of physical activity, but its partial reflection only. Fourth, there was a lack of information on dietary habits [40], which could contribute to the development of dyslipidemia. Thus, we failed to eliminate the risk of residual confounding by dietary habits. Fifth, we did not collect data on insulin resistance and blood glucose level. These are considered possible sources of bias when investigating the associations between CRF and blood lipids [41]. Lastly, external validity must not be sufficiently high. As our study participants were urban Japanese women with an interest in a physical fitness test, we cannot claim that they are representative of the entire population of adult Japanese women. In addition, the median follow-up period was shorter than that of previous studies [21]. Therefore, this study warrants further research with longer follow-up period including Japanese men and other ethnic groups and races with different lifestyles as participants.

## 5. Conclusions

Our study indicates a significant association of cardiorespiratory fitness and BMI with the incidence of dyslipidemia among Japanese women. We conclude that maintaining a high cardiorespiratory fitness level and normal weight is important for the prevention of dyslipidemia among Japanese women.

## Figures and Tables

**Table 1:** Characteristics based on cardiorespiratory fitness at baseline.

Characteristics	Unit	All (n = 927)	Cardiorespiratory Fitness Level			*p*-Value
			Lowest Tertile (n = 314)	Middle Tertile (n = 309)	Highest Tertile (n = 304)	
Person-years		1665	531	545	589	
Follow-up years	years	1.0 (1.0,16.0)	1.0 (1.0,16.0)	1.0 (1.0,14.0)	1.0 (1.0,14.0)	0.385
PWC75% HR Max	watt/kg	1.60 (0.54)	1.14 (0.33)	1.59 (0.31)	2.08 (0.49)	<0.001
Number of cases		196	86	56	54	0.005
Age	years	48.9 (16.2)	49.3 (16.4)	49.0 (16.1)	48.5 (16.2)	0.827
Body mass index	kg/m^2^	22.2 (3.9)	24.0 (4.5)	21.8 (3.5)	20.7 (2.6)	<0.001
Systolic BP	mmHg	110.9 (16.3)	114.1 (16.7)	111.4 (15.2)	108.2 (16.5)	<0.001
Diastolic BP	mmHg	63.9 (10.4)	66.4 (10.5)	63.2 (10.2)	62.2 (10.2)	<0.001
Pulse pressure	mmHg	47.0 (10.4)	47.8 (10.8)	47.0 (9.8)	46.2 (10.6)	0.156
Total cholesterol	mg/dL	201.2 (21.2)	201.7 (27.7)	201.1 (28.9)	200.9 (30.4)	0.946
LDL cholesterol	mg/dL	104.8 (21.2)	109.3 (19.7)	103.7 (21.5)	101.4 (21.6)	<0.001
HDL cholesterol	mg/dL	68.1 (15.0)	63.8 (13.3)	69.0 (15.5)	71.3 (15.2)	<0.001
Triglycerides	mg/dL	68.9 (26.4)	74.9 (27.4)	68.3 (25.7)	63.7 (24.9)	<0.001
Smokers	%	6.9	5.7	5.8	9.2	0.252
Drinkers	%	37.2	33.1	35.0	43.8	0.018

Data are presented as mean (standard deviation), median (min, max), or percentages. PWC 75% HR Max, peak work capacity 75% heart rate max; BP, blood pressure; LDL, low-density lipoprotein; HDL, high-density lipoprotein.

**Table 2:** Hazard ratios with 95% confidence intervals for the incidence of dyslipidemia based on cardiorespiratory fitness and body mass index.

	**Cardiorespiratory Fitness Level**				***p* for Trend**
	**Lowest Tertile** **(n = 314)**	**Middle Tertile** **(n = 309)**		**Highest Tertile** **(n = 304)**	
No. of cases per 1000 person-years	162.0	102.8		91.7	
Age-adjusted HRs (95% CI)	1.00 (reference)	0.67 (0.48–0.94)		0.63 (0.45–0.89)	0.006
Multivariable HRs (95% CI) ^1^	1.00 (reference)	0.69 (0.49–0.97)		0.66 (0.47–0.94)	0.017
Multivariable HRs (95% CI) ^2^	1.00 (reference)	0.87 (0.61–1.24)		0.97 (0.66–1.42)	0.83
	**Body Mass Index**				***p*-Value**
	**<25 kg/m^2^ (n = 738)**		**≥25 kg/m^2^ (n = 188)**		
No. of cases per 1000 person-years	87.4		275.1		
Age-adjusted HRs (95% CI)	1.00 (reference)		2.73 (2.36–3.66)		<0.001
Multivariable HRs (95% CI) ^1^	1.00 (reference)		2.63 (1.94–3.55)		<0.001
Multivariable HRs (95% CI) ^2^	1.00 (reference)		2.54 (1.84–3.50)		<0.001

^1^ Adjusted for age plus alcohol drinking, smoking, systolic blood pressure. ^2^ Adjusted for ^1^ plus body mass index or cardiorespiratory fitness. HRs, hazard ratios; CI, confidence interval.

**Table 3:** Joint analysis of hazard ratios with 95% confidence intervals for the incidence of dyslipidemia based on body mass index and cardiorespiratory fitness.

	Cases Per 1000 Person-Years	Age-Adjusted HRs (95% CI)	Multivariable- HRs (95% CI) ^1,2^	Joint HRs (95% CI) ^2^
**BMI ≥ 25 kg/m^2^**				
Lowest CRF tertile (n = 64)	230.7	1.00 (reference)	1.00 (reference)	1.00 (reference)
Middle CRF tertile (n = 63)	280.8	1.12 (0.64–1.97)	1.20 (0.67–2.17)	1.19 (0.67–2.10)
Highest CRF tertile (n = 61)	328.9	1.23 (0.70–2.16)	1.36 (0.75–2.48)	1.43 (0.80–2.53)
**BMI < 25 kg/m^2^**				
Lowest CRF tertile (n = 253)	109.0	1.00 (reference)	1.00 (reference)	0.54 (0.33–0.89)
Middle CRF tertile (n = 248)	82.8	0.80 (0.52–1.21)	0.80 (0.52–1.22)	0.43 (0.25–0.73)
Highest CRF tertile (n = 238)	70.4	0.69 (0.44–1.07)	0.70 (0.45–1.09)	0.38 (0.22–0.65)

^1^*p* for interaction (CRF category by BMI category) < 0.01; ^2^ Adjusted for age, alcohol drinking, smoking, and systolic blood pressure. BMI, body mass index; CRF, cardiorespiratory fitness; HRs, hazard ratios; CI, confidence interval.
